# Advances and Challenges in the Management of Myelodysplastic Syndromes

**DOI:** 10.3390/cancers17152469

**Published:** 2025-07-25

**Authors:** Jessica M. Stempel, Tariq Kewan, Amer M. Zeidan

**Affiliations:** Section of Medical Oncology and Hematology, Department of Internal Medicine, Yale School of Medicine, Yale Comprehensive Cancer Center, New Haven, CT 06510, USA

**Keywords:** myelodysplastic syndromes, myelodysplastic neoplasms, MDS, precision medicine, risk stratification, hypomethylating agents

## Abstract

Myelodysplastic syndromes/neoplasms (MDS) represent a biologically and clinically diverse group of myeloid malignancies marked by cytopenias, morphological dysplasia, and an inherent risk of progression to acute myeloid leukemia. Over the past two decades, the field has made significant advances in characterizing the molecular landscape of MDS, leading to refined classification systems to reflect the underlying genetic and biological diversity. In 2025, the treatment of MDS is increasingly individualized, guided by integrated clinical, cytogenetic, and molecular risk stratification tools. For lower-risk MDS, the treatment paradigm has evolved beyond erythropoiesis-stimulating agents (ESAs) with the introduction of novel effective agents such as luspatercept and imetelstat, as well as shortened schedules of hypomethylating agents (HMAs). For higher-risk disease, monotherapy with HMAs continue to be the standard of care as combination therapies of HMAs with novel agents have, to date, failed to redefine treatment paradigms. The recognition of precursor states like clonal hematopoiesis of indeterminate potential (CHIP) and the increasing use of molecular monitoring will hopefully enable earlier intervention/prevention strategies. This review provides a comprehensive overview of the current treatment approach for MDS, highlighting new classifications, prognostic tools, evolving therapeutic options, and ongoing challenges. We discuss evidence-based recommendations, treatment sequencing, and emerging clinical trials, with a focus on translating biological insights into improved outcomes for patients with MDS.

## Simple Summary

Myelodysplastic syndromes/neoplasms (MDS) represent a group of diverse hematological malignancies that lead to bone marrow failure, low blood counts, and a variable risk of progression to acute myeloid leukemia. Over the last two decades, significant insights have been gained into the underlying biology of MDS, including the contribution of genetic mutations, immune dysregulation, and the bone marrow microenvironment to disease pathogenesis. By summarizing recent studies, therapeutic developments, and evolving treatment algorithms through the lens of precision medicine, this review aims to guide clinicians in delivering personalized, risk-adapted care for individuals with lower- and higher-risk MDS, to discuss the unmet needs and main challenges and to inform future research directions in this rapidly advancing field.

## 1. Introduction

Myelodysplastic syndromes/neoplasms (MDS) represent a heterogeneous group of clonal myeloid malignancies characterized by ineffective hematopoiesis, cytopenias, and an inherent risk of progression to acute myeloid leukemia (AML). MDS predominantly affects the older population, with an incidence rate of 26.1 per 100,000 in individuals aged 65 and older and a median age of diagnosis of 77 years [1,2]. Clonal cytopenias of undetermined significance (CCUS), MDS, and AML exist along a continuum defined by diverse morphological, cytogenetic, and molecular features and varying risks of morbidity and mortality. Over the past several years, interest in MDS has grown exponentially, driven mainly by a deeper understanding of the role of the bone marrow microenvironment and immune dysregulation in its pathogenesis [3,4], as well as by advancements in molecular characterization and its impact on prognostication. These insights have fueled the pursuit of novel therapeutic strategies and innovative drug combinations, addressing a critical need for improved treatment options in this population. This progress builds on several decades of the evolution of MDS management from its initial morphological classification in the 1980s to today’s personalized approaches, reflecting substantial advances in diagnosis, risk stratification, and treatment. We aim to comprehensively review the current management and future directions and challenges in treating patients with MDS.

### 1.1. Challenges in Classification and Risk-Assessment: The Evolving System(s) for MDS

The classification of MDS has evolved from exclusive reliance on morphology-based criteria to incorporating molecular data that better correlate with prognosis [5,6,7]. In 2022, two working groups revised and published novel classifications for MDS: the World Health Organization (WHO) Classification [6] and the International Consensus Classification (ICC) [5]. Both systems aimed to capture the heterogeneity and complexity of MDS and reflect recent advances in understanding the biology of the disease. However, the coexistence of these two parallel systems leads to ambiguity among pathologists, clinicians, and patients, complicating clinical trial design and affecting the interpretation and applicability of research findings. A notable divergence is the introduction by the ICC of an “AML/MDS” category, defined by 10–19% circulating and/or bone marrow blasts (excluding cases with AML-defining cytogenetics and ≥10% blasts) [5]. Additionally, both classification systems now recognize *TP53*-mutated MDS as a distinct, higher-risk entity (‘MDS with biallelic *TP53* inactivation’ in WHO and ‘myeloid neoplasms with *TP53*’ in ICC) [5,6]. Supporting the emphasis on molecular classification, Bernard et al. demonstrated that blast percentage alone did not universally predict the outcome among molecular subgroups, suggesting that genetically-defined subtypes should drive future MDS classification approaches [8,9]. Furthermore, recent findings from the International Consortium for MDS (icMDS) demonstrated significant molecular heterogeneity within morphologically-defined MDS cases, with specific mutations correlating distinctly with clinical phenotype and prognosis [10]. Collectively, these advancements advocate for a greater reliance on molecular signatures to enhance diagnostic precision, prognostic accuracy, and therapeutic decision making, including the expansion of more intensive therapies and clinical trials that mirror the aggressiveness of the disease phenotype [11,12].

The field would greatly benefit from the unification of these systems into a single, consensus-based classification framework. Such an alliance should integrate emerging knowledge of the biology, prognosis, and novel technological advances. For an in-depth review of the classification systems and proposed synchronization strategies, the reader is directed to an expert report by the icMDS [13].

### 1.2. Risk-Assessment in the Era of Precision Medicine

The prognosis of MDS varies significantly depending on the patient’s age, degree of cytopenias, percentage of bone marrow blasts, and cytogenetic abnormalities. The International Prognostic Scoring System (IPSS) [14] and revised IPSS (IPSS-R) [15] are well established and validated tools widely used in clinical practice and clinical trials to assess disease risk, inform treatment decisions, and assess trial eligibility. However, these prognostic tools fall short of accounting for the increasingly relevant genetic landscape. The newly developed molecular IPSS (IPSS-M) incorporates key genetic alterations and offers a more comprehensive risk stratification reflecting the robust association of gene mutations on prognosis [16]. The IPSS-M has been validated in multiple studies, including in a higher risk cohort of patients treated with hypomethylating agents (HMA) [17], and performs well in patients with LR-MDS and those treated with an allogeneic hematopoietic stem cell transplant (allo-HCT) [18]. As the prognostic significance of additional, rarer, and currently less well-defined genetic alterations becomes clearer, the scoring systems will likely continue to evolve and become more precise and individualized.

Lower risk MDS (LR-MDS) is commonly defined as having low or intermediate-1 risk groups according to IPSS (score ≤ 1); very low, low, or some patients with intermediate risk groups by IPSS-R (score ≤ 3.5); and very low, low, and moderate low under IPSS-M (score ≤ 0). In contrast, higher risk MDS (HR-MDS) includes individuals with scores exceeding these thresholds. We will adhere to these definitions throughout the review.

### 1.3. Treatment of Lower-Risk MDS

The primary treatment goal for individuals with LR-MDS is to improve and/or preserve quality of life by reducing transfusion burden and complications associated with cytopenias. Arguably, treatment also should aim to cure the disease and prolong survival as patient outcomes within the “lower risk” category can be highly variable. The median overall survival (OS) for IPSS-R very low, low, and intermediate risk is 8.8, 5.3, and 3 years, respectively [15], with similar duration for low and moderate low IPSS-M (4.6–6 years) [16].

Erythropoiesis-stimulating agents (ESAs) remain a mainstay of therapy for LR-MDS and are discussed in detail in later sections. Novel therapies for LR-MDS have primarily benefited patients with anemia as the predominant cytopenia. In contrast, treatment options for those with clinically significant isolated thrombocytopenia and/or neutropenia remain limited and rely most on supportive care measures or hypomethylating agents (HMA). Immunosuppressive-based therapy can be considered for selected patients with hypoplastic MDS [19], HLA-DR15 histocompatibility type, or a paroxysmal nocturnal hemoglobinuria clone, similar to the management of acquired aplastic anemia [20]. Thrombopoietin agonists, such as eltrombopag [21] and romiplostim [22], have been investigated in this population given their ability to stimulate not only platelet count recovery but also trilineage hematopoiesis, as observed in patients with aplastic anemia [23]. While they have been shown to reduce bleeding events, they carry a higher incidence of grade 3 and 4 adverse events. A recent study showed no difference in clonal evolution with eltrombopag compared to placebo in patients with LR-MDS; however, a theoretical risk of accelerated progression to AML remains a concern when these agents are used in HR-MDS [21,24]. Asymptomatic patients who do not require platelet or red blood cell (RBC) transfusions and have an absolute neutrophil count > 500 cell/microL may be managed with a “watch and wait” approach, with laboratory monitoring at regular intervals based on the degree of cytopenias and risk of progression. Supportive care is essential for all patients with MDS, especially those with profound cytopenias which impact quality of life and progression-free survival (PFS) [25,26]. An extensive review on supportive care for patients with MDS is beyond the scope of this paper [27].

Allo-HCT is generally not recommended as the initial treatment strategy for LR-MDS as delayed transplant at disease progression or clonal evolution is associated with improved gain in survival [28,29]. Some exceptions could include patients who have profound and/or refractory cytopenias or who have an underlying germline predisposition to myeloid malignancies [30].

LR-MDS and CCUS, defined as persistent (>4 months) and unexplained cytopenias with evidence of clonal hematopoiesis in the absence of morphological dysplasia, share some overlapping clinical and molecular features [5]. Xie et al. demonstrated that high-risk CCUS is associated with clinical outcomes comparable to those of LR-MDS and reported that both PFS and OS are similar to those observed in LR-MDS [31]. Recent efforts have led to the development of clinical calculators such as the Clonal Hematopoiesis Risk Score (CHRS) [32] and the Clonal Cytopenia Risk Score (CCRS) [31]. These models incorporate molecular features, including number of mutations and variant allele frequency, to estimate the risk for progression to myeloid neoplasms. The CHRS, derived from large population datasets, stratifies clonal hematopoiesis of indeterminate potential and CCUS into low-, intermediate-, and high-risk categories, with the high-risk group demonstrating a markedly elevated 10-year cumulative incidence for myeloid neoplasm of 52.2%. In contrast, the CCRS was developed exclusively in CCUS cohorts utilizing three adverse factors: presence of splicing factor mutations, number of mutations (≥2), and thrombocytopenia (<100 × 10^9^/L), to predict the 2-year risk of progression to AML/MDS (Table 1). Both scoring systems offer practical frameworks for identifying high-risk individuals with CCUS that may behave biologically akin to LR-MDS who may benefit from closer surveillance, early intervention, or inclusion in clinical trials typically reserved for patients with LR-MDS. These risk models exemplify the shift toward precision risk assessment in the evaluation of clonal cytopenias.

### 1.4. MDS with Chromosome 5 Deletion

The management of MDS with chromosome 5 deletion (del[5q]) is distinct from other subtypes of MDS as it is highly responsive to lenalidomide [33]. A multicenter phase II study demonstrated that 76% (95% confidence interval [CI], 68–82) of patients had a reduction in the number of transfusions while 67% (95% CI, 59–74) became transfusion independent (median duration of response not reached after 2.2 years of follow-up) [33]. A subsequent phase III study showed a significant improvement in the RBC transfusion independence (RBC-TI) [34] rate among patients treated with lenalidomide 10 mg (61%) and 5 mg (51.1%), compared to the placebo cohort (7.8%) (*p* < 0.001), as well as an AML-free survival advantage [35]. Among patients who are RBC-TI, Díez-Campelo et al. reported that an early intervention with low dose lenalidomide (5 mg daily) significantly reduced the risk for RBC transfusion dependence by nearly 70% (hazard ratio [HR] = 0.203, 95% CI: 0.132–0.692, *p* = 0.0046) [36]. The short and long-term benefits of lenalidomide support maximizing the treatment duration in this subgroup of MDS. Nevertheless, the response often is ultimately lost and alternate therapies such as ESAs if erythropoietin [EPO] ≤ 500 mU/mL, HMAs, imetelstat, luspatercept, or clinical trials if EPO > 500 mU/mL can be considered [20].

### 1.5. Management of Anemia in LR-MDS

**Figure 1:**
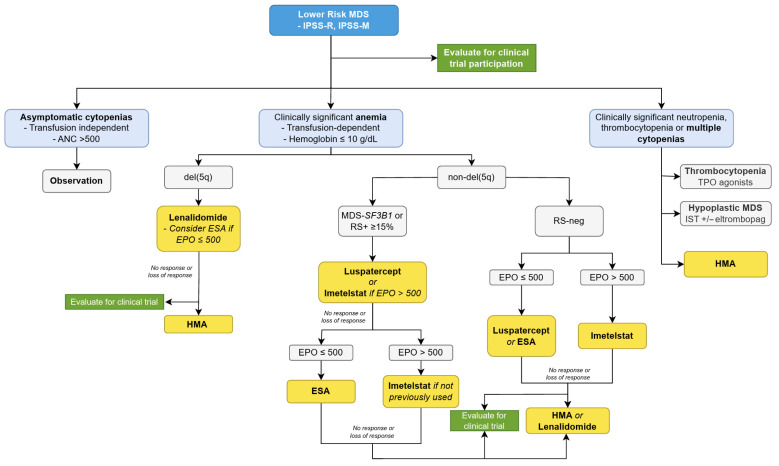
A proposed treatment algorithm of patients with LR-MDS in 2025. Footnotes: ANC: absolute neutrophil count, EPO: erythropoietin, ESA: erythropoietin-stimulating agents, HMA: hypomethylating agents, IST: immunosuppressive therapy, RS: ringed sideroblasts, TPO: thrombopoietin.

The majority of patients with MDS will develop anemia during the course of their disease, and approximately 50% will require RBC transfusions. Traditionally, ESAs have been considered the first-line treatment for patients with LR-MDS and clinically significant anemia (hemoglobin < 10 g/dL or requiring RBC transfusions). ESAs effectively reduce RBC transfusion needs, particularly in patients with low endogenous EPO levels and lower transfusion requirements [37,38]. However, the quality of response is variable, and most patients will relapse to their transfusion-dependent state approximately 2 years after starting treatment [39]. Patients that relapse early experience an increased risk for AML progression and a shorter OS. These implications have driven the exploration of newer agents such as luspatercept and imetelstat (Figure 1).

### 1.6. Luspatercept

Luspatercept is a transforming growth factor-beta superfamily ligand trap which enhances late-stage erythropoiesis and ameliorates MDS-related anemia [40]. Luspatercept is currently approved for the treatment of anemia in patients with LR-MDS who are ESA-naïve and may require RBC transfusion [41]. Luspatercept is also approved for the treatment of LR-MDS and MDS/myeloproliferative neoplasm with ring sideroblasts and thrombocytosis (MDS/MPN-RS-T) with RBC transfusion-dependent (RBC-TD) anemia who have relapse/refractory disease [41].

The phase II PACE-MDS trial included patients with LR-MDS who were RBC-TD and previously treated with ESAs [42]. The study demonstrated erythroid hematological improvement (HI-E) in two thirds of patients, with 38% achieving RBC-TI, regardless of baseline EPO level or prior ESA use [42]. Patients with the lowest transfusion requirements had earlier and more sustained improvement in hemoglobin levels (median increase, 2 g/dL). Additionally, a robust response was seen in patients with MDS with ≥15% ringed sideroblasts (MDS-RS; RBC-TI 42%, HI-E 69%) and MDS with an *SF3B1* mutation (RBC-TI 44%, HI-E 77%). Long term results of the PACE-MDS support the use of luspatercept for non-RS MDS which have a nearly 36% HI-E rate [43]. The phase 3 MEDALIST trial, conducted exclusively in patients with MDS with RS ≥ 15% and RS ≥ 5% with an *SF3B1* mutation who were RBC-TD and refractory or ineligible to ESAs, showed that 38% of patients on luspatercept achieved RBC-TI for at least 8 weeks over a 24-week period, compared to 13% in the placebo group (*p* < 0.001) [44].

Frontline luspatercept was compared to epoetin alpha in ESA-naïve and RBC-TD patients in the phase 3 randomized COMMANDS trial [45]. The RBC-TI rate (TI for ≥12 weeks and hemoglobin increase ≥ 1.5 g/dL) was significantly greater with luspatercept compared to ESA (59 vs. 31%, *p* < 0.001), and correlated with significant improvement in health-related quality of life metrics [46]. Long-term results further confirmed the superiority of luspatercept, with a longer median duration of RBC-TI (35.6 months versus 20.9 months with ESA; *p* = 0.0016) and a positive OS trend [47], bringing luspatercept to the frontline of RBC-TD LR-MDS [48]. The benefit was irrespective of RS status, although only 26% were RS-negative in this trial. The observed trend towards improved OS with luspatercept with longer follow up of the COMMANDS trial raises the possibility that its role may extend beyond supportive care and warrants a consideration of potential natural history modifying effects. Luspatercept is being compared to ESA in the randomized ELEMENT-MDS trial in patients with LR-MDS who have symptomatic anemia (hemoglobin ≤ 9.5 g/dL) but are RBC-TI and ESA-naïve (NCT05949684) [49].

Real-world data on the use of luspatercept are also emerging to allow treatment patterns in the clinical setting to be better understood. A recent analysis shows that the recommended dose-escalation schedule of luspatercept is underutilized which may limit its full therapeutic potential and reduce the proportion of patients achieving optimal responses [50]. Additional studies exploring initiating luspatercept at the maximum dose (1.75 mg/kg) have shown favorable results without new safety signals [51,52].

### 1.7. Imetelstat

Imetelstat is a first-in-class direct and competitive telomerase inhibitor approved for the treatment of patients with RBC-TD LR-MDS who have relapsed after, are refractory to, or are ineligible for ESAs [53]. Approval was based on results from the randomized phase III IMerge trial which showed significantly higher rates of durable RBC-TI at multiple time points, including 8 weeks, 24 weeks and 1 year, in the imetelstat group compared to placebo [54]. The benefit was observed across RS status and baseline transfusion requirements. Notably, results suggested that imetelstat carried disease-modifying activity, evidenced by cytogenetic responses, the reduction in malignant clonal populations, and correlation with longer durations of response. The impact of the perceived disease modification on survival will need to be seen with longer follow-up. More recent data shows that imetelstat retains clinical activity regardless of prior treatments for LR-MDS (including luspatercept) and prior response to ESA [55]. Although this analysis was limited by a small sample size, the findings inform drug sequencing practices in the evolving treatment landscape of LR-MDS. Imetelstat offers an alternative for patients who do not respond to or are ineligible for ESAs (EPO > 500 mU/mL) with the potential to delay progression and next-line of therapy. This mechanism sets it apart from other agents that primarily target erythropoiesis without addressing the underlying clonal dynamics of MDS.

RBD-TD is associated with reduced quality of life metrics and inferior clinical outcomes, particularly in those who develop secondary iron overload [26,56,57]. Iron overload can lead to end-organ damage if left untreated, including hepatic siderosis and iron-overload cardiomyopathy, which can further complicate prognosis and management. Iron chelation therapy plays an important role in mitigating these risks. Current treatment guidelines recommend starting iron chelation therapy for patients who have received more than 20 units of RBC transfusions and a ferritin level exceeding 2500 ng/mL, aiming for levels < 1000 ng/mL [20,27,58].

### 1.8. HMAs in LR-MDS

Treatment with HMAs earlier in the disease’s course has garnered growing interest due the potential to modify the natural history of MDS. Unlike traditional supportive care, HMAs such as azacitidine (AZA) and decitabine (DEC) target epigenetic dysregulation and promote hematological improvement. Prospective studies have shown that attenuated HMA dosing can lead to meaningful hematological responses, including improvement in anemia and transfusion requirements, and a favorable toxicity profile [59]. Patients with relapsed or refractory MDS, with ongoing RBC transfusion requirements, and with elevated EPO levels should be considered for a clinical trial or more intensive HMA-based treatments [20].

The availability of these newer therapies for LR-MDS introduces a welcome complexity in determining the optimal selection, sequencing, and combination of therapies. While ESAs remain an important option for patients with low EPO and the absence of RS, luspatercept in our opinion is the preferred first-line treatment for all patients with non-del5q LR-MDS with clinically significant isolated anemia (Figure 1). Other novel agents with different mechanisms of action that target anemia in LR-MDS are listed in Table 2. As the therapeutic landscape continues to evolve, ongoing studies and real-world evidence are critical to clarifying the most effective strategies for integrating these novel agents into the management of LR-MDS in clinical practice.

### 1.9. Treatment of Higher-Risk MDS

**Figure 2:**
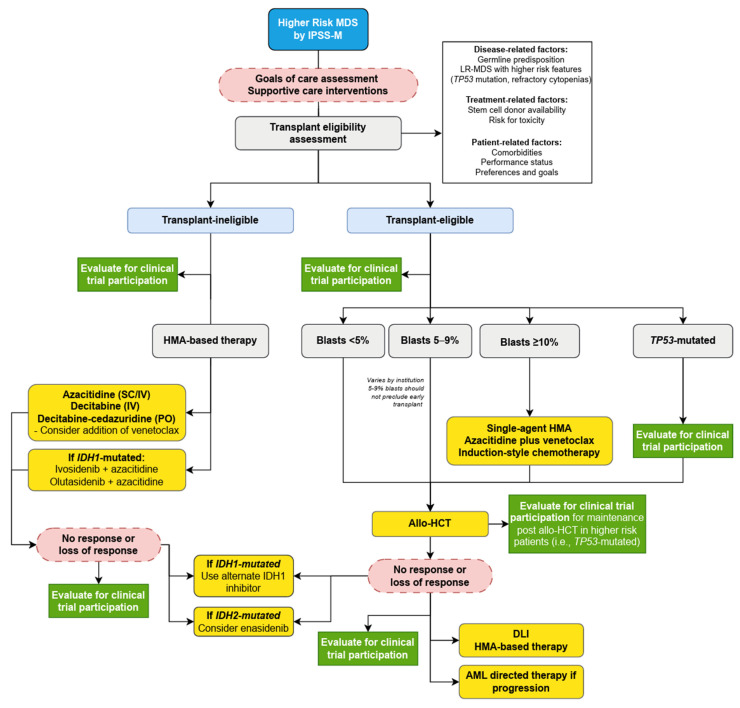
A proposed treatment algorithm of patients with HR-MDS in 2025. Footnotes: Allo-HCT: allogeneic stem cell transplant, DLI: donor lymphocyte infusion HMA: hypomethylating agents, IV: intravenous, SC: subcutaneous.

Despite the rapid expansion of treatment options for LR-MDS, HR-MDS continues to represent a significant unmet clinical need. The median OS for patients with IPSS-R high and very high risk is 1.6 and 0.8 years [15], respectively, with similar predictions under the IPSS-M (1.7 and 1.0 years, respectively) [16], and therefore the main goal of treatment in HR-MDS is to delay leukemic transformation and extend survival. Allo-HCT remains the only potentially curative treatment option; however, the associated morbidity and mortality risks limit patient eligibility, necessitating separate treatment approaches for transplant-eligible and -ineligible individuals (Figure 2).

### 1.10. Transplant Eligible Patients

HMA-based therapy is the standard of care in HR-MDS, ideally used as a bridging tool to allo-HCT. Individuals with significant frailty, comorbidities, poor performance status, and advanced age (>80 years) are generally considered ineligible for transplant [71]. Studies have demonstrated that patients with HR-MDS and a readily available suitable donor for allo-HCT experience better outcomes, supporting its strong consideration for all eligible patients regardless of age [72,73,74]. Detailed discussion regarding donor selection, conditioning, and transplant logistics are increasingly complex and beyond the scope of this review, and the reader is referred to excellent recent reviews on this subject [75]. For example, there are increasing data that haploidentical donors and mismatched unrelated donors can be used successfully in allogeneic transplantation for MDS patients when combined with post-transplantation cyclophosphamide [76,77]. We do, however, emphasize the need for the early referral of any potentially eligible patient with higher-risk MDS for transplantation consideration/consultation.

Intensive induction therapy as a bridge to transplant should be considered for patients with an elevated bone marrow blast count (≥10%) or lacking a suitable donor [71]. The recommendation for cytoreduction for ≥10% blasts is based on the positive correlation between higher blast count at the time of transplant and relapse risk [71,78,79]. Guidance on intensive induction for blasts between 5 and 9% is less clear. In practice, these patients are considered to have HR-MDS and are candidates for early transplant, but whether there is added benefit of intensive induction or HMA therapy to achieve morphological CR before allo-HCT is still debated. Schroeder et al. reported that patients who underwent immediate allo-HCT had a significantly higher 5-year OS probability compared to those who received pre-transplant therapy (68.5% vs. 37.4%, *p* = 0.023), with similar results for those with 5–9% blasts [80]. A large systematic review found that achieving post-induction CR before transplant did not correlate with longer OS [81]. Furthermore, a large cohort dataset of HMA-treated patients reported that achieving a composite CR, using the new International Working Group 2023 criteria [82], correlated with a superior OS (HR = 0.602, *p* < 0.001) only in patients who did not undergo transplantation [83]. These findings highlight the uncertainly regarding the role of bridging, and prospective trials are needed to determine which patients and disease features would benefit most from cytoreduction.

Post-transplant maintenance in MDS remains an area of active investigation. There is particular interest in patients with *TP53* mutations who stand to gain the greatest benefit from novel approaches given their poor outcomes even after allo-HCT. In a large trial for patients with AML and MDS, there was no difference in relapse free survival (RFS) between maintenance subcutaneous AZA compared to placebo [84]. Low-dose DEC (5 mg/m2 days 1–5, 28-day cycle) plus recombinant human G-CSF was shown to reduce the incidence of AML relapse post-transplant in a randomized phase II trial of 204 patients with AML [85]. Notably, the study included only a small number of patients harboring *TP53* mutations (n = 4). The randomized phase III AMADEUS trial (NCT04173533) [86] of oral AZA vs. placebo maintenance is currently underway after showing early safety and efficacy results [87]. Other maintenance approaches are being explored and are reported in Table 3.

Measurable residual disease (MRD) monitoring may be a more sensitive gauge of disease burden than morphology alone and should be routinely assessed and validated in prospective clinical trials [96]. Monitoring patient-specific MRD after transplant in MDS is challenging but has the potential to identify early relapse, predict transplant response, and select patients for post-transplant maintenance or pre-emptive therapies [97,98,99,100].

### 1.11. Transplant-Ineligible

HMAs are the cornerstone of front-line therapy for transplant ineligible patients since the approval of AZA in 2004 [20,101]. AZA, DEC, and oral decitabine-cedazuridine (C-DEC) are the only FDA-approved first-line agents for HR-MDS and form the foundation of many emerging combination strategies (Table 4). Despite their widespread use, HMAs offer limited OS benefit, highlighting the need for more effective treatment options.

The pivotal phase III AZA-001 trial demonstrated a superior OS of 24.5 months with AZA (75 mg/m^2^) compared to 15 months with best available therapy [115]. The results of this study likely overestimate survival outcomes, as real-world data have not consistently replicated such a robust response with a median OS up to around 18 months [116,117]. DEC was evaluated in a randomized phase III trial which showed a significantly superior overall response rate (ORR) and statistically non-significant trends towards longer time to AML progression and OS [118]. Approximately 20% of patients who receive HMA and reach a stable disease response can accomplish a better response at a later timepoint during treatment [119]. In practice, patients should continue therapy until disease progression or unacceptable toxicity or they are on the path to allo-HCT. Recently, oral C-DEC showed equivalent pharmacological features and efficacy as intravenous decitabine, and a median OS of 31.8 months [120,121]. This study lacked a comparator arm, limiting our ability to assess clinical superiority to intravenous DEC.

Although most patients respond within the initial 4–6 cycles of HMA (efficacy assessment), unfortunately most will ultimately relapse; therefore, newly diagnosed patients with HR-MDS should be strongly considered for a clinical trial whenever possible.

### 1.12. Early Promise and Later Disappointments: HMA Combinations in HR-MDS

Multiple novel HMA combination strategies have failed to yield successful results in large clinical trials despite promising early results.

The combination of AZA with the histone deacetylase inhibitor vorinostat suggested high response rates in early-phase studies [122]. However, the randomized phase II trial (SWOG S1117) found no OS benefit and a higher drug discontinuation rate in the vorinostat plus AZA arm [123]. Similarly, AZA and pevonedistat, a NEDD8-activating enzyme inhibitor, showed synergy with AZA in preclinical models, and early studies showed favorable outcomes [124]. The subsequent phase III PANTHER trial showed no significant improvement in event-free survival (EFS) or OS with the combination [106]. Magrolimab, an anti-CD47 macrophage checkpoint inhibitor, also showed encouraging results and high response rates [125] The following phase III ENHANCE trial was terminated early as an interim analysis showed no OS advantage [126]. Lastly, the later phase trials of AZA plus sabatolimab (STIMULUS-MDS1 and 2), an anti-T-cell receptor T-cell immunoglobulin and mucin domain-3 (TIM-3) antibody, failed to meet their primary endpoint despite early promise [108,127,128]. These results underscore the heterogeneity of HR-MDS and the challenges of improving survival in this population. Novel combination trials are ongoing and anticipated to help address the significant unmet need in this highly heterogenous and complex patient population.

Venetoclax, a potent small molecule BCL-2 inhibitor, reshaped the treatment paradigm for older or unfit patients with AML. The landmark VIALE-A trial demonstrated a significant improvement in median OS, nearly doubling survival with the addition of venetoclax to AZA when compared to AZA alone (14.7 versus 9.6 months) [129]. Stemming from the success of this combination in AML and the observed synergy in myeloid malignancies [130], several studies have emerged in MDS [131,132].

In the front-line setting, real-world data showed the efficacy of the AZA/venetoclax combination for patients with HR-MDS, with an emphasis on better outcomes for patients who are able to reach transplant [133]. One recent study demonstrated superior CR rates with HMA/venetoclax compared to HMA alone (33% vs. 12%, *p* < 0.001) but found no difference in OS [134]. A phase 1b study published in 2025 by Garcia et al. reported that 30% of patients with treatment-naïve HR-MDS reached a CR as best response with AZA/venetoclax and had a median OS of 26 months, which was similar among patients who achieved CR and mCR with HI [135]. These results are encouraging and superior to historical responses with AZA monotherapy. Additionally, nearly 40% were able to proceed to allo-HCT and had a median OS not reached (NR, 95% CI: 40.1-NR). Unsurprisingly, survival was shorter in the *TP53*-mutated population (11.2 months). Longer follow-up is needed to confirm if patients have experienced prolonged responses and a survival benefit. The highly anticipated global phase III randomized VERONA trial aimed to assess the efficacy and safety of AZA/venetoclax (AZA 75 mg/m2 IV or SC; venetoclax 400 mg daily for 14 days; 28-day cycles) versus AZA monotherapy in treatment-naïve HR-MDS [136]. The trial did not meet its primary endpoint of OS (HR 0.908, *p* = 0.3772) according to a recent press release, and further details are awaited to fully interpret these findings and their clinical implications [102]. Clinical and molecular data, rates of allo-HCT, and subsequent therapies will be important for interpreting the study outcomes and assessing the full clinical impact of the AZA/venetoclax combination. The combination of AZA/venetoclax is increasingly used in clinical practice for transplant-ineligible individuals; however, key questions remain regarding patient selection and optimal dosing and the scheduling of therapy. These uncertainties highlight the need for additional studies to better define effective consolidation and maintenance strategies in this setting.

In addition, the combination of C-DEC and venetoclax was also recently explored, and after a median follow-up of 10.8 (IQR 5.6–16.4) months, 37 of 39 patients responded, and nearly half underwent allo-HCT, highlighting the potential role of these regimens as a bridge to transplant [137].

It is important to note that venetoclax is associated with significant and prolonged myelosuppression, which in clinical practice can lead to treatment delays, dose reductions, and life-threatening bleeding and infectious complications. Patients who are on the path to transplant could be considered for this intensive approach to reduce blast count pre-transplant given the reported rapid responses observed with this combination [131].

### 1.13. IDH1/2 Inhibitors

*IDH1/2* mutations are present in approximately 5% of patients with MDS and are associated with a higher progression to AML [138].

The IDH1 inhibitor, ivosidenib (IVO), is currently approved for relapsed/refractory *IDH1*-mutated MDS based on the AG120-C-001 MDS sub-study results which showed CR rates of nearly 40% and a median OS of 35.7 months [113]. The IDIOME study, a single arm phase II by the GFM, demonstrated favorable results with IVO [139,140]. Among patients with previous HMA failure, the study reported an ORR of 63.6%, and a median OS of 8.9 months. In treatment-naïve HR-MDS, the addition of AZA to IVO after three cycles led to an ORR of 78.3%, with a median OS not reached after 25.2 months of follow-up. The global PyramIDH phase III randomized trial comparing single-agent HMA and single-agent IVO in the front-line setting is currently underway (NCT06465953) [141].

The IDH1 inhibitor olutasidenib is currently approved for the treatment of relapsed/refractory *IDH1*-mutated AML [142]. Olutasidenib led to durable remissions in patients with HR-MDS (n = 22) in a multicenter, open-label, phase I/II trial [143] and continues to be explored in combination with HMAs in an ongoing phase II trial (NCT06597734) [144].

Enasidenib (ENA), an oral selective IDH2 inhibitor, is approved for the treatment of relapsed or refractory *IDH2*-mutated AML and has demonstrated promising results in MDS. An open-label phase I trial conducted by Stein et al. reported an ORR of 53% (nine of 17 patients). A favorable response was also seen in six of 13 patients who were previously treated with HMA (ORR 46%) [145]. A subsequent phase II trial, which included 50 patients with MDS, evaluated the safety and efficacy of AZA plus ENA combination in a treatment-naïve cohort and ENA monotherapy for patients pre-treated with HMA. The majority of patients treated with the combination achieved a response (ORR 74%) with a median time to best response of 1.3 (0.9–3.8) months, whereas 52% reached stable disease with ENA monotherapy (ORR 35%) and experienced a slower median time to best response of 4.6 (2.7–7.6) months [146].

### 1.14. TP53-Mutated MDS

Mutations in *TP53* leads to a loss of the normal tumor suppressive functions and promotes genomic instability and disease progression. In MDS, these mutations identify a very high-risk subgroup within HR-MDS characterized by an aggressive disease biology and resistance to conventional therapies. Patients with MDS with *TP53* mutations, particularly those with bi-allelic inactivation, have a notably poor prognosis and are at high risk for AML transformation [147]. Responses to conventional HMA and HMA-based therapies are often brief, making allo-HCT the preferred strategy when feasible.

Despite its urgent clinical need, *TP53*-mutated MDS has remained largely refractory to therapeutic advances. APR-246 (eprenetapopt), a first-in class small molecule designed to restore wild-type p53 function, initially generated excitement based on early-phase data suggesting responses when combined with AZA [148,149]. However, enthusiasm was tempered by the results of the pivotal phase III trial which failed to meet its primary endpoint of CR when comparing AZA plus APR-246 to AZA monotherapy [150]. Similarly, the phase Ib magrolimab plus AZA study included 25 patients (26%) with a *TP53* mutation, of whom 10 achieved CR and a median OS of 16.3 months (95% CI, 1.8-NR) [125]. Nevertheless, subsequent data failed to show a clinical benefit in this high-risk population [126]. The phase Ib sabatolimab plus HMA trial reported an ORR of 71.4% (95% CI, 41.9–91.6) among 10 of 14 *TP53*-mutated HR-MDS patients (four of whom reached CR, 28.6%) [128]. However, the phase III STIMULUS-MDS2 trial failed to meet its primary endpoint of OS [127].

Effective and durable therapies for *TP53*-mutated MDS remain an urgent unmet need. Emergent treatment approaches and clinical trial enrollment should be strongly recommended for this subgroup of individuals with high-risk MDS biology.

## 2. Conclusions

The current management of MDS is dynamic and rapidly evolving, driven by advances in molecular diagnostics, refined classification systems, and the promise of targeted therapies. The approach to treatment is risk-adapted and biologically informed, aiming to match therapeutic intensity with disease severity, molecular profile, and individual patient goals. Given the complexity of MDS and its treatment landscape, a multidisciplinary and collaborative approach, ideally involving specialized pathologists and hematologists at centers with expertise in myeloid malignancies, is essential to deliver optimal care, provide access to emerging therapies, and facilitate participation in clinical trials. Continued collaborative research remains critical to translating the biological complexity of MDS into durable and meaningful clinical benefit.

## Figures and Tables

**Table 1:** Comparison of Risk Stratification Models for Clonal Cytopenias: CHRS vs. CCRS.

Feature	CHRS (Clonal Hematopoiesis Risk Score)	CCRS (Clonal Cytopenia Risk Score)
Publication	Weeks et al., NEJM Evidence, 2023 [32]	Xie et al., Blood, 2024 [31]
Purpose	Predicts risk of progression to myeloid neoplasm malignancy in patients CHIP/CCUS	Stratifies CCUS patients based on progression risk to MDS or AML
Population	CHIP and CCUS (including individuals without cytopenias)	Specifically developed for CCUS
Key Components	8 prognostic factors: Number of mutations, specific genes (high-risk mutations, *DNMT3A*), VAF, presence of cytopenia, RDW, MCV, age	3 prognostic factors: Number of mutations (≥2), specific genes (splicing factor mutations), and platelet count < 100 × 10^9^/L
Risk Scoring	Low risk: ≤9.5 points Intermediate risk: 10–12 points High risk: ≥12.5 points	Low risk: <2.5 points Intermediate risk: 2.5–≤5 points High risk: ≥5 points
Gene weighting	Higher risk for splicing factor mutations, AML-like (*IDH1/2*, *FLT3*, *RUNX1*), *JAK2* and *TP53* assigned score 2.5; if ≥2 mutations assigned score of 2	Splicing factor mutations assigned score of 2; if ≥2 mutations assigned score of 3
Risk for MN	5-year CI for MN (±SD) (%): No CHIP/CCUS: 0.0740 (±0.0064) Low risk: 0.232 (±0.0484) Intermediate risk: 2.76 (±0.482) High risk: 24.4 (±4.12)	2-year CI (%, 95% confidence interval) of MN progression: Low risk: 6.4 (13–11.4) Intermediate risk: 14.1 (7.9–22.2) High risk: 37.2 (19.8–54.7)
Strengths	Large population cohort; integrates age and VAF; validated	Only 3 prognostic factors; CCUS-specific; validated

AML: acute myeloid leukemia, CCUS: clonal cytopenias of undetermined significance, CHIP: clonal cytopenias of indeterminate potential, CI: cumulative incidence, MDS: myelodysplastic syndromes, MN: myeloid neoplasms, SD: standard deviation, VAF: variant allele frequency.

**Table 2:** Selected Ongoing Clinical Trials of LR-MDS.

Agent or Drug Combination	Mechanism of Action	Trial Phase	Population	Clinical Trial ID	References
Luspatercept vs. ESA	TGF-β superfamily ligand trap	III	LR-MDS (IPSS-R ≤ 3.5), RBC-TI	NCT05949684	Zeidan et al., ASH, 2023 [49]
KER-050 (Elritercept)	Modified activin receptor type IIA ligand trap	II	LR-MDS (IPSS-R ≤ 3.5)	NCT04419649	Diez-Campelo et al., ASH, 2023 [60]; Tan et al., ASH, 2024 [61]
RVU120	CDK8/19 small molecule inhibitor	II	LR-MDS (IPSS-R ≤ 3.5), R/R or ineligible for other therapies	NCT06243458 [62]	ClinicalTrials.gov
Canakinumab	Anti-IL-1β human monoclonal antibody	II	LR-MDS (IPSS-R ≤ 3.5), R/R ESA, HMAs	NCT04239157	Rodriguez Sevilla et al., ASH, 2023 [63]
Attenuated durations of HMAs	DNMT inhibitor	II	LR-MDS (IPSS ≤ 1)	NCT02269280	Bouligny et al., EHA, 2025 [64]
Luspatercept + ESA	TGF-β superfamily ligand trap	I/II	LR-MDS (IPSS ≤ 1), non-RS, R/R ESA	2021-000596-37 *	Ades et al., ASH, 2024 [65]
Oral decitabine + cedazuridine (ASTX727)	DNMT inhibitor	I/II	LR-MDS (IPSS ≤ 1), non-RS, R/R	NCT03502668	Garcia-Manero et al., SOHO, 2023 [66]
LB-100	Protein phosphatase 2A inhibitor	Ib/II	LR-MDS (IPSS ≤ 1), requiring treatment	NCT03886662 [67]	ClinicalTrials.gov
R289	IRAK1/4 inhibitor	Ib	LR-MDS (IPSS-R ≤ 3.5), R/R, included del(5q)	NCT05308264	Garcia-Manero et al., ASH, 2024 [68]
DFV890	Selective NLRP3 inhibitor	Ib	LR-MDS (IPSS-R ≤ 3.5), previously treated	NCT05552469	Garcia-Manero et al., ASH, 2024 [69]
SX-682 +/− Decitabine	CXCR1/2 inhibitor	I	LR-MDS (IPSS ≤1), R/R	NCT04245397 [70]	ClinicalTrials.gov

CKD: cyclin-dependent kinase, CXCR4: CXC chemokine receptor 4, DNMT: DNA methyltransferase, ESA: erythropoiesis stimulating agent, HMAs: hypomethylating agents, IL: interleukin, IPSS: international prognostic scoring system, IPSS-R: IPSS-revised, IRAK: interleukin1 receptor-associated kinase, LR-MDS: lower risk MDS, MDS: myelodysplastic syndromes, NLRP3: nucleotide-binding and leucine-rick repeat P3, R/R: relapsed/refractory, RBC-TI: red blood cell transfusion independent, TGF-ß: transforming growth factor-beta. * European Union clinical trials register identifier.

**Table 3:** Maintenance therapy trials for patients who remain in remission after transplant.

Agent/Drug Combination	Trial Phase	Status	Population	Results	Clinical Trial ID	Reference
Oral azacitidine vs. placebo	III	Active	AML, MDS	N/A	NCT04173533	ClinicalTrials.gov [86]
IL-2	I/Ib	Active	AML, MDS	N/A	NCT06138587	ClinicalTrials.gov [88]
Decitabine and cedazuridine	I/II	Recruiting	MDS	N/A	NCT04742634	ClinicalTrials.gov [89]
IDH1 inhibitor	I	Completed	AML, MDS	CIR 19% (95% CI: 4.0–41) 2y-PFS 81% (95% CI: 52–94) 2y-OS 88% (95% CI: 59–97)	NCT03564821	Fathi et al., Clin Cancer Res, 2023 [90]
IDH2 inhibitor	I	Completed	AML, MDS	CIR 16% (95% CI: 3.7–36) 2y-PFS 69% (95% CI: 39–86) 2y-OS 74% (95% CI: 44–90)	NCT03515512	Fathi et al., Blood Adv, 2022 [91]
Eprenetapopt + azacitidine	II	Completed	*TP53*-mutated AML, MDS	1-y PFS 59.9% (95% CI: 41–74) 1y-OS 78.8% (95% CI: 60.6–89.3) MRD monitoring after transplant predicts outcome	NCT03931291	Mishra et al., JCO, 2022 [92]
Azacitidine + DLI	II	Completed	AML, MDS	2y-PFS 68.3% (95% CI: 58.3–80.1) 2y-OS 76% (95% CI:52–90)	NCT01541280	Guillaume, et al., Transpl and Cel Ther, 2021 [93]
Panobinostat	I/II	Phase III Terminated	AML, MDS	Phase II results: CIR 20% (95% 7–33) 2y-RFS 75% (63–90). 2yr-OS 81% (95% CI: 69–95)	NCT04326764 [94]	Bug et al., Leukemia, 2017 [95]

AML: acute myeloid leukemia, CI: confidence interval, CIR: cumulative incidence rate of relapse, DLI: donor lymphocyte infusion, IDH: isocitrate dehydrogenase, IL: interleukin, MDS: myelodysplastic syndromes, MPN: myeloproliferative neoplasms, OS: overall survival, PFS: progression-free survival.

**Table 4:** Selected Clinical Trials of HR-MDS.

Agent/Drug Combination	Trial PHASE	Target/MOA	Population	Results	Status	Clinical Trial ID
Venetoclax + AZA [102]	III	BCL-2 inhibitor + DNMTi	HR-MDS	Did not meet primary endpoint of OS; HR 0.908, *p* = 0.3772	Active, not recruiting	NCT04401748
APR-246 (Eprenetapopt) + AZA [103]	III	*TP53*	*TP53*-mutant HR-MDS	APR-246 + AZA arm: CR 34.6% AZA arm: CR 22.4%	Completed	NCT03745716
Rigosertib vs. BSC [104]	III	Microtubule-destabilizing agent	HR-MDS after failure of HMAs	Rigosertib: OS 8.2 months (95% CI: 6.1–10.1) Best supportive care: OS 5.9 months (95% CI: 4.1–9.3) (HR 0.87, 95% CI: 0.67–1.14; *p* = 0.33)	Completed	NCT02562443
AZA and Cedazuridine (ASTX030) [105]	Multi-phase	Oral DNMTi	MDS, CMML, AML	NA	Active, recruiting	NCT04256317
AZA + pevonedistat [106]	III	NEDD8-activating enzyme	HR-MDS, CMML or AML with 20–30% blasts	Pevonedistat + AZA arm: median EFS 17.7 months AZA arm: median EFS 15.7 months (HR 0.968; 95% CI: 0.757–1.238; *p* = 0.557) in the HR-MDS cohort	Completed	NCT03268954
AZA + magrolimab [107]	III	Anti-CD47 monoclonal antibody + DNMTi	HR-MDS	Magrolimab + AZA arm: CR 21.3% AZA arm: CR 23.6%	Terminated	NCT04313881
AZA + sabatolimab [108]	III	TIM-3 inhibitor + DNMTi	MDS, CMML-2	Median PFS: Sabatolimab + AZA arm: 11.1 months AZA arm: 8.5 months (*p* = 0.102) CR rate: Sabatolimab + AZA arm: 21.5% AZA arm: 17.7% (*p* = 0.769)	Terminated	NCT04266301
AZA + tamibarotene [109]	III	(RARα) agonist + DNMTi	HR-MDS	Tamibarotene + AZA arm: CR 23.8% AZA arm: CR 18.8% (*p* = 0.2084)	Terminated	NCT04797780
AZA + Lisaftoclax [110]	III	BCL-2 inhibitor + DNMTi	HR-MDS	NA	Recruiting	NCT06641414
AZA + AK117 [111]	I/II	Anti-CD47 monoclonal antibody + DNMTi	HR-MDS	NA	Active, not recruiting	NCT04900350
AZA +/− BGB-11417 [112]	I/II	BCL-2 inhibitor + DNMTi	MDS, AML	NA	Recruiting	NCT04771130
Ivosidenib [113]	I	IDH1 inhibitor +/− DNMTi	Hematologic malignancies with *IDH1* mutations	ORR of 83.3% and CR rate of 38.9% in 18 patients	Active, recruiting	NCT02074839
Enasidenib [114]	II	IDH2 inhibitor +/− DNMTi	R/R MDS, HR-MDS, LR-MDS resistant to ESA	Enasidenib monotherapy (cohort A):best OR 42.9% after 3–6 cycles	Active, not recruiting	NCT03744390

AZA: azacitidine, AML: acute myeloid leukemia, BSC: best supportive care, DNMTi: DNA methyltransferase inhibitor, CI: confidence interval, CMML: chronic myelomonocytic leukemia, CR: complete remission, EFS: event free survival, ESA: erythropoiesis-stimulating agents, HMA: hypomethylating agents, HR: hazard ratio, HR-MDS: higher risk myelodysplastic syndrome, LR-MDS: lower risk MDS, MOA: mechanism of action, NA: not available, OR: overall response, ORR: overall response rate, OS: overall survival, PFS: progression free survival, R/R: relapse refractory, TIM-3: T-cell immunoglobulin domain and mucin domain-3.
